# Tumor-infiltrating plasma cells are a prognostic factor in penile squamous cell carcinoma

**DOI:** 10.1007/s00428-024-04013-1

**Published:** 2025-01-14

**Authors:** P. J. Stenzel, A. Thomas, M. Schindeldecker, S. Macher-Goeppinger, S. Porubsky, A. Haferkamp, I. Tsaur, W. Roth, K. E. Tagscherer

**Affiliations:** 1 Institute of Pathology, University Medical Center Mainz, Langenbeckstr. 1, 55131 Mainz, Germany; 2 Dr. Senckenberg Institute of Pathology, University Medical Center Frankfurt, Theodor-Stern-Kai 7, 60590 Frankfurt am Main, Germany; 3 Department of Urology, University Medical Center Mainz, Langenbeckstr. 1, 55131 Mainz, Germany; 4 Department of Urology, University Hospital Tübingen, Hoppe-Seyler-Str. 3, 72076 Tuebingen, Germany

**Keywords:** Tumor microenvironment, Tumor-infiltrating plasma cells, Penile carcinoma, Prognosis, Immunotherapy

## Abstract

Penile cancer (PeCa) is a rare disease with poor prognosis in the metastatic stage. Neither effective adjuvant nor palliative therapeutic options are available. Research efforts in this field have so far failed to establish robust predictors of survival. To identify prognostic targets in PeCa, the current project focused on characterizing the tumor microenvironment (TME). A study cohort of 93 men with PeCa was used for the construction of a tissue microarray and immunohistochemical staining for CD3, CD4, CD8, CD20, CD56, CD138, FoxP3, and PD-L1. The quantity and spatial distribution of tumor-infiltrating immune cells were analyzed using digital image analysis. PD-L1 staining of tumor and immune cells was manually scored (combined positivity score (CPS)). T cells, T helper cells, cytotoxic T cells (CTLs), and regulatory T cells were detected in > 90% of PeCa and B cells in 88%, plasma cells in 85%, and NK cells in 23%. Approximately 50% of the PeCa samples were PD-L1 positive. In the univariate survival analysis, high PD-L1 CPS, plasma cells, CTLs, and B cells were significantly associated with favorable overall survival (OS), and the latter two with adverse recurrence-free survival. In multivariate analysis, plasma cells remained a significant factor for favorable OS (*p* = 0.04). In this study, the immune cells in the TME, especially plasma cells, were favorably associated with patient survival compared to other established prognostic factors in PeCa. Contemporarily, plasma cells have been discussed in the light of contributing to responses to modern immunotherapies. The results of this study support this notion.

**Supplementary Information:**

The online version contains supplementary material available at 10.1007/s00428-024-04013-1.

## Introduction

Penile cancer (PeCa) is a rare disease with 26,000 cases per year worldwide [1]. Infection with human papilloma virus (HPV), especially the high-risk subtypes 16 and 18, phimosis and smoking are associated with an increased risk of PeCa [2]. Histologically, PeCa is a squamous cell carcinoma (SCC) of the usual type in most cases. Other subtypes, such as basaloid SCC, are predominant in men with HPV infections. During the clinical course, PeCa can infiltrate the cavernous bodies and access the lymphatic system or blood vessels with the risk of lymphatic or distant dissemination. While patients with localized disease have an excellent prognosis with a 5-year disease-specific survival (DSS) of 95%, these rates for patients with advanced stages range between 80 and 35%, depending on the extent of lymph node involvement, and 16% for patients with distant metastasis [3]. Penile-sparing surgery or other local treatment options are the standard of care for localized tumor stages, with a focus on optimal oncological and functional outcomes [4]. The treatment of choice for locally advanced disease is partial or radical penectomy, combined with surgical staging of the groins. In metastatic PeCa, state-of-the-art therapy encompasses platinum-based chemotherapy protocols [4].

Since PeCa is a disease with a low incidence, evidence for optimal treatment strategies in different therapy settings is limited, and there is an urgent need for novel treatment regimens as well as reliable predictive and prognostic parameters. HPV status is important for risk stratification since patients with HPV-associated tumors usually exhibit a better prognosis and might benefit from HPV vaccination in combination with immune checkpoint inhibitors (ICIs) [5]. Other promising treatment options include targeted therapies and ICI [5–7]. In recent years, the tumor microenvironment (TME) with tumor-infiltrating immune cells and the expression of immune checkpoint receptors of both tumor and immune cells have gained increasing attention owing to their prognostic value for patient survival and response to therapy.

There are limited data on the impact of tumor-infiltrating immune cells and PD-L1 on the prognosis of patients with PeCa. Thus, the aim of this study was to analyze the content and spatial distribution of immune cells in the TME of the PeCa cohort of the University Medical Center Mainz, and to correlate the density of these cells with clinical outcomes and clinicopathological parameters.

## Methods and materials

### Study cohort

The study cohort comprised 93 men with non-metastatic invasive squamous cell carcinoma of the penis who underwent surgical treatment at the Department of Urology of the University Medical Center of the Johannes Gutenberg University Mainz between 1996 and 2019, as previously described [6, 7]. Briefly, all patients received primary surgery without prior systemic treatment and the consecutive disease management adhered to the currently valid European Association of Urology (EAU) Guidelines on Penile Cancer. Comprehensive information on clinical patient characteristics, such as age at diagnosis, HPV status, treatment, and survival, and pathological tumor features, such as tumor grade and stage was collected. All tumors were re-staged according to the 8th edition of the TNM classification [8]. The HPV-Status was assessed through PCR-based sequencing as previously described [6, 7]. Locally advanced tumors were defined as pathological disease states pT3 and pT4. Disease recurrence was defined as previously described [6, 7]. The study protocol was designed in accordance with the principles of the Declaration of Helsinki. The use of excess tissue samples older than 4 years was approved, and the need for written informed consent from the patients was waived by the Ethics Committee of the State Medical Association of Rhineland-Palatinate because of the retrospective design of the study (ethics approval number: 837.360.16 (10,679)).

### Tissue microarray construction and immunohistochemistry

All patients with available formalin-fixed paraffin-embedded PeCa tissue samples were included, and the tissue microarray (TMA) was constructed as previously described [6]. The TMA slides were immunohistochemically stained for CD3 (IR503, Dako, Glostrup, Denmark), CD4 (4B12, Dako), CD8 (C8/144B, Dako), CD20 (IR604, Dako), CD56 (123C3, Dako), CD138 (MI15, Dako), FoxP3 (236A/E7, Abcam, Cambridge, United Kingdom), and PD-L1 (EPR19759, Abcam) using automated immunostainers (autostainer plus, Dako).

### Digital image analysis

All slides were digitalized using a digital whole-slide scanner (Nanozoomer, Hamamatsu Photonics, Hamamatsu, Japan) and analyzed using the HALO® platform (Indica Labs, Corrales, NM, USA). Digital image analysis was performed as previously described [9–11]. Briefly, the TMA cores were manually annotated to differentiate between the tumor parenchyma and tumor-associated stroma. The size of the respective areas and biomarker-positive cells were quantified using detection algorithms implemented in HALO®. These algorithms can automatically differentiate between stain-positive and stain-negative cells after manual adjustment of the thresholds. A representative set of cores was used to define the analysis settings and thresholds for each stain. To avoid distortion of the results, small areas with strong artificial overlap and detritus were manually excluded from the analysis by annotation layers. Missing or erroneous TMA cores were excluded from analysis. The settings of the analysis algorithms were validated on a set of randomly selected cores, especially with regard to the false detection of aberrantly stained cells (e.g., tumor cell positivity for CD138) and the results were checked for plausibility. The final immune cell density was calculated as follows: (1) immune cell density in the tumor parenchyma = number of immune cells in the tumor parenchyma/mm^2^ tumor parenchyma, (2) immune cell density in the tumor-associated stroma = number of immune cells in the tumor-associated stroma/mm^2^ tumor-associated stroma, (3) total immune cell density = number of immune cells in the tumor parenchyma and associated stroma/mm^2^ tumor parenchyma and associated stroma.

### PD-L1 scoring

PD-L1 expression was manually scored. The tumor proportion score (TPS) is the proportion of PD-L1 positive tumor cells to total tumor cells, the combined positivity score (CPS) is the proportion of PD-L1 positive tumor cells and associated immune cells to total tumor cells, and the immune cell score (IC) is the proportion of the area of positive immune cells to the total tumor area.

### Statistical analysis

For dichotomization, the Charité Cutoff finder [12] was used to distinguish between low and high expression levels based on patient survival data. Overall survival (OS), recurrence-free survival (RFS), and disease-specific survival (DSS) were defined as previously described [6, 7]. The survival curves were depicted by Kaplan–Meier plots, and the significance of differences was calculated with the log-rank test. The Cox regression model was used for the univariate and multivariate analyses. The association of the immune cell densities with the clinicopathological parameters was tested for significance using the Mann–Whitney-Wilcoxon test. Differences were considered significant when the error probability was less than 0.05.

## Results

### Patient collective

A total of 93 patients with PeCa with a mean age at diagnosis of 65.0 years were included in the study cohort (Table 1). The median age at diagnosis was 67 years (range 31–90 years). Eight (8.6%) patients underwent circumcision, four (4.3%) underwent tumor excision, 55 (59.1%) underwent partial penectomy, and 25 (26.9%) underwent total penectomy. A total of 21 (22.6%) patients had high-grade tumors, 27 (29.0%) had locally advanced tumor stages, 22 (23.7%) had lymph node metastases, and 24 (25.8%) had associated HPV infection (Table 1).

**Table 1:** Patient cohort

	*N*	%
**Cohort size**	93	100
**Age at diagnosis**		
≤ 65 years	44	47.3
> 65 years	49	52.7
**Primary tumor** **surgery**		
Circumcision	8	8.6
Tumor excision	4	4.3
Partial penectomy	55	59.1
Total penectomy	25	26.9
Missing	1	1.1
**Histologic subtype**		
Usual type	74	79.6
Basaloid	15	16.1
Missing	4	4.3
**Tumor grading**		
G1/G2	70	75.3
G3/G4	21	22.6
Missing	2	2.2
**Tumor stage**		
pT1	35	37.6
pT2	27	29.0
pT3	27	29.0
Missing	4	4.3
**Lymph node metastasis**		
N0/Nx	60	64.5
N1	7	7.5
N2	6	6.5
N3	9	9.7
Missing	11	11.8
**Recurrence status**		
No	78	83.9
Yes	15	16.1
**Subsequent** **therapy**		
None	67	72
CTX	16	17.2
Radiation	1	1.1
Unknown	6	6.5
CTX and radiation	2	2.2
Missing	1	1.1
**HPV infection**		
Negative	68	73.1
Positive	24	25.8
Missing	1	1.1
**Tumor-dependent** **death**		
No	81	87.1
Yes	12	12.9

*N* number, *CTX* chemotherapy

### Immunohistochemistry of tumor-infiltrating immune cells and PD-L1

Cytotoxic T cells (CTL) were present in all analyzed PeCa samples (91/91 analyzed tumors). Regulatory T cells (Tregs) were detected in 88 PeCa (88/93 analyzed tumors), T cells in 87 PeCa (87/89 analyzed tumors), T helper cells (Th cells) in 82 PeCa (82/89 analyzed tumors), B cells in 80 PeCa (80/91 analyzed tumors), plasma cells in 78 PeCa (78/92 analyzed tumors), and natural killer cells (NK cells) in 21 PeCa (21/92 analyzed tumors). In the spatial analysis, there were higher mean and median densities of all analyzed immune cell subtypes in the tumor-associated stroma than in the tumor parenchyma, except for NK cells. Approximately one-third of all analyzed PeCa samples were positive for PD-L1, as assessed by the TPS (31/90 analyzed tumors) or the IC (33/90 analyzed tumors). Approximately half of the tumors were PD-L1 positive using the CPS (48/90 analyzed tumors). The results are presented in Table 2.

**Table 2:** PD-L1 scores and densities and distribution of immune cells in penile cancer

Immune cells (biomarker)	PD-L1 score/compartment	Total *N*	NA *N*	Pos. cases *N* (%)	Min	Median	Max	Mean	STD
	TPS	90	3	31 (34.4)	0	0	80.0	5.4	13.5
PD-L1	CPS	90	3	48 (53.3)	0	0.5	90.0	6.5	14.0
	IC	90	3	33 (36.7)	0	0	10.0	0.8	1.8
	Total	88	5	87 (98.9)	0	729.758	4634.22	1036.7	1028.4
T cells	Tumor	88	5	87 (98.9)	0	496.381	3398.86	788.25	791.64
(CD3)	Stroma	88	5	87 (98.9)	0	1502.46	5033.41	1805.2	1400.6
	Total	89	4	82 (92.1)	0	70.6173	4049.4	218.93	489.9
T helper cells	Tumor	89	4	82 (92.1)	0	40.2607	1379.91	109.64	186.17
(CD4)	Stroma	88	5	79 (89.8)	0	131.99	4849.17	448.86	744.71
	Total	91	2	91 (100)	5.9	283.5	3221.4	505.7	620.7
CTL	Tumor	90	3	90 (100)	5.1	146.6	3601.5	398.9	616.4
(CD8)	Stroma	88	5	88 (100)	10.5	508.0	2964.4	750.2	727.2
	Total	91	2	80 (87.9)	0	20.7	1144.8	107.5	214.2
B cells	Tumor	93	2	65 (69.9)	0	2.4	619.4	38.4	116.9
(CD20)	Stroma	89	4	73 (82.0)	0	58.9	3052.7	238.7	494.6
	Total	92	1	21 (22.8)	0	0	323.2	11.7	47.9
NK cells	Tumor	93	0	16 (17.2)	0	0	468.8	14.3	65.5
(CD56)	Stroma	91	2	17 (18.7)	0	0	512.4	13.1	58.1
	Total	92	1	78 (84.8)	0	24.3	1829.4	157.1	316.5
Plasma cells	Tumor	91	2	25 (27.5)	0	0	208.4	4.3	22.5
(CD138)	Stroma	87	6	73 (83.9)	0	112.3	5687.5	671.4	1112.1
	Total	93	0	88 (94.6)	0	22.2	628.1	57.6	104.0
Treg	Tumor	93	0	83 (89.2)	0	9.3	934.8	33.4	104.6
(FoxP3)	Stroma	93	0	86 (92.5)	0	76.4	2155.3	199.2	324.8

*N* number, *NA* not analyzed, *Pos*. positive, *Min* minimum, *Max* maximum, *STD* standard deviation, *PD-L1* programmed death receptor ligand 1, *CTL* cytotoxic T cells, *NK cells* natural killer cells, *Treg* regulatory T cells, *TPS* tumor proportion score, *CPS* combined proportion score, *IC* immune cells

### Association of tumor-infiltrating immune cells and PD-L1 with clinicopathological parameters

Patients who died from any cause had lower densities of CTL and tumor-infiltrating B cells (TIL-B) (*p* ≤ 0.05) as well as lower PD-L1 CPS and IC (*p* ≤ 0.05). A higher density of Th cells and CTL was present in the tumor-associated stroma (*p* ≤ 0.05) of patients with disease relapse and a lower PD-L1 CPS and IC (*p* ≤ 0.05) or lower TIL-B (*p* ≤ 0.05) in patients with lymph node metastases or distant metastases, respectively. HPV positivity was associated with higher densities of tumor-infiltrating Th cells and NK cells and a higher PD-L1 IC score compared to HPV-negative PeCa (*p* ≤ 0.05). Patients with associated lichen sclerosus had a higher density of tumor-infiltrating NK cells (*p* ≤ 0.01) and Tregs (*p* ≤ 0.05), and patients with associated balanoposthitis had a higher density of TIL-B (*p* ≤ 0.05). Pre-existing conditions were associated with altered densities of tumor-infiltrating immune cells, such as adiposity with higher TIL-B (*p* ≤ 0.05) and Tregs (*p* ≤ 0.05), smoking with lower NK cells (*p* ≤ 0.05) and Tregs (*p* ≤ 0.05), and diabetes mellitus with a lower density of tumor infiltrating T cells as well as a lower PD-L1 TPS (*p* ≤ 0.05). The results are presented in Table 3.

**Table 3:** Association of tumor infiltrating immune cells with clinical parameters

	*N*	Mean ± STD	*N*	Mean	
Overall survival status	Alive		Deceased		*p* value
PDL1 CPS	61	7.57 ± 14.1	26	4.75 ± 14.44	< 0.05
PDL1 IC	61	1.03 ± 2.1	26	0.15 ± 0.34	< 0.05
CD8 total	60	612.96 ± 712.79	28	310.04 ± 306.2	< 0.05
CD20 tumor	59	55.03 ± 142.32	29	7.47 ± 16.92	< 0.05
**Disease relapse**	**Absent**		**Present**		
CD4 stroma	70	451.56 ± 744.92	13	797.5 ± 798.04	< 0.05
CD8 stroma	71	706.93 ± 693.24	14	1080.46 ± 866.32	< 0.05
**Lymph nodemetastasis**	**Absent**		**Present**		
PDL1 CPS	55	7.95 ± 14.34	22	4.91 ± 15.49	< 0.05
PDL1 IC	55	1.11 ± 2.19	22	0.12 ± 0.28	< 0.05
**Distant metastasis**	**Absent**		**Present**		
CD20 tumor	24	30.41 ± 119.98	4	0.38 ± 0.44	< 0.05
**HPV**	**Absent**		**Present**		
PDL1 IC	66	0.7 ± 1.89	21	0.99 ± 1.53	< 0.05
CD4 total	63	233.56 ± 544.66	21	270.99 ± 344.21	< 0.05
CD4 tumor	63	87.14 ± 189.83	21	157 ± 172.9	< 0.05
CD56 tumor	67	12.61 ± 67.15	22	21.85 ± 67.29	< 0.05
CD56 total	67	8.78 ± 40	22	22.11 ± 68.95	< 0.05
**Lichen sclerosus**	**Absent**		**Present**		
CD56 stroma	84	12.88 ± 60.05	4	28.39 ± 29.81	< 0.01
CD56 total	84	11.67 ± 49.88	5	18.91 ± 20.08	< 0.01
FoxP3 stroma	84	182.09 ± 323.5	5	446.8 ± 330.44	< 0.05
**Balanoposthitis**	**Absent**		**Present**		
CD20 tumor	79	27.52 ± 85.28	9	143.22 ± 262.54	< 0.05
**Adipositas**	**Absent**		**Present**		
CD20 total	75	100.97 ± 218.82	13	150.84 ± 210.25	< 0.05
CD20 tumor	75	23.79 ± 79.84	13	129.17 ± 229.51	< 0.05
FoxP3 tumor	76	20.28 ± 41.12	13	41.1 ± 60.98	< 0.05
**Smoking**	**Absent**		**Present**		
CD56 stroma	65	18.37 ± 68.14	23	0.05 ± 0.22	< 0.05
CD56 total	66	16.23 ± 56.02	23	0.16 ± 0.76	< 0.05
FoxP3 stroma	66	235.55 ± 365.41	23	86.25 ± 133.87	< 0.01
FoxP3 total	66	58.99 ± 96.57	23	26.9 ± 45.25	< 0.05
**Diabetes mellitus**	**Absent**		**Present**		
PDL1 TPS	70	6.73 ± 14.97	17	0.88 ± 2.64	< 0.05
CD3 total	68	1240.55 ± 1066.88	15	647.92 ± 525.95	< 0.05

*N* number, *STD* standard deviation

### Survival analysis

**Fig. 1:**
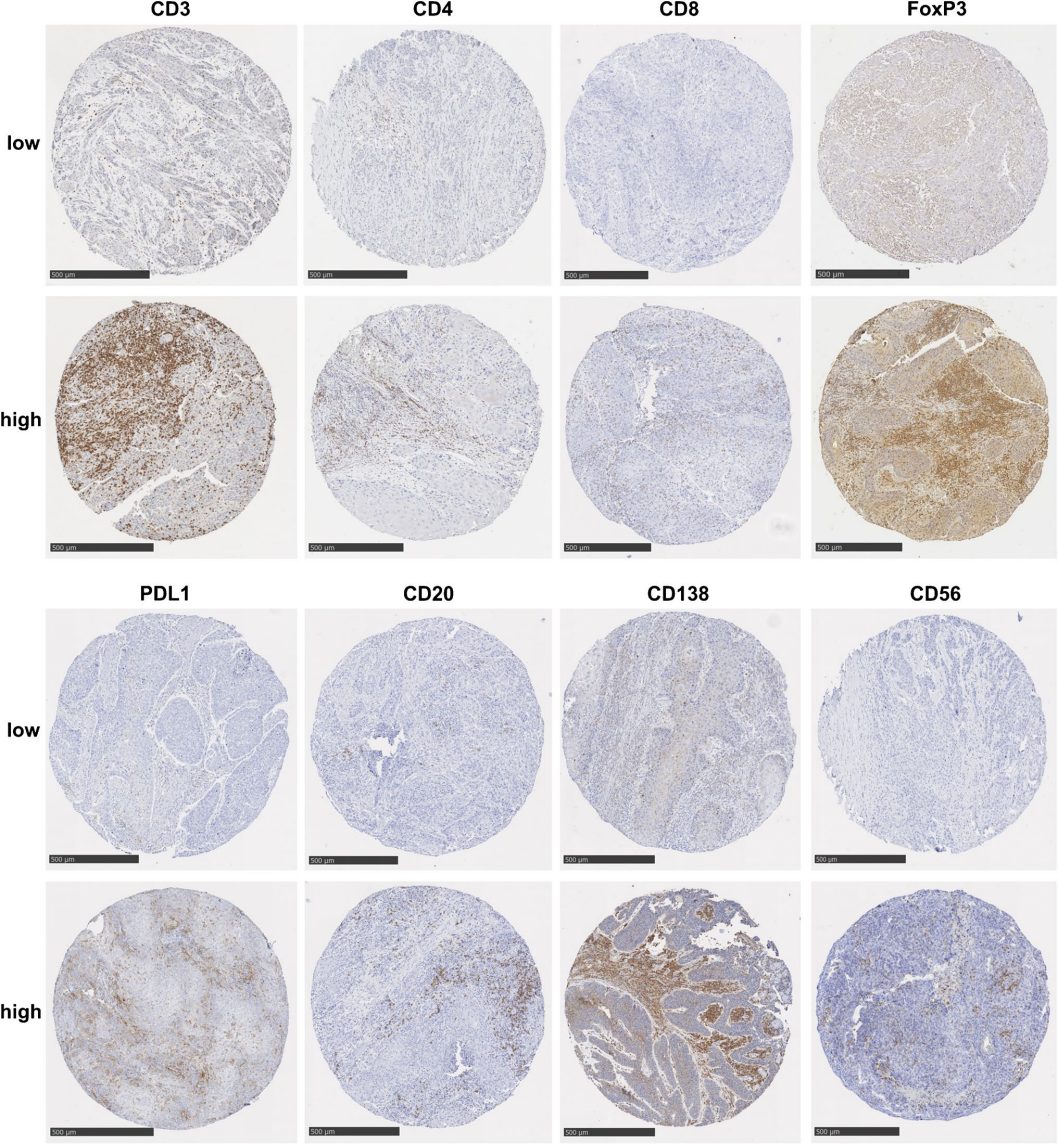
Tumor-infiltrating immune cells and PD-L1 in penile cancer (PeCa). All samples of the patient cohort were immunohistochemically stained for PD-L1 expression, CD3 for detection of T cells, CD4 for T helper cells, CD8 for cytotoxic T cells, FoxP3 for regulatory T cells, CD20 for B cells, CD138 for plasma cells, and CD56 for natural killer cells. The patient cohort was then dichotomized into tumors with low or high immune cell infiltrate or PD-L1 score, respectively. Scale bar represents 500 µm

**Fig. 2:**
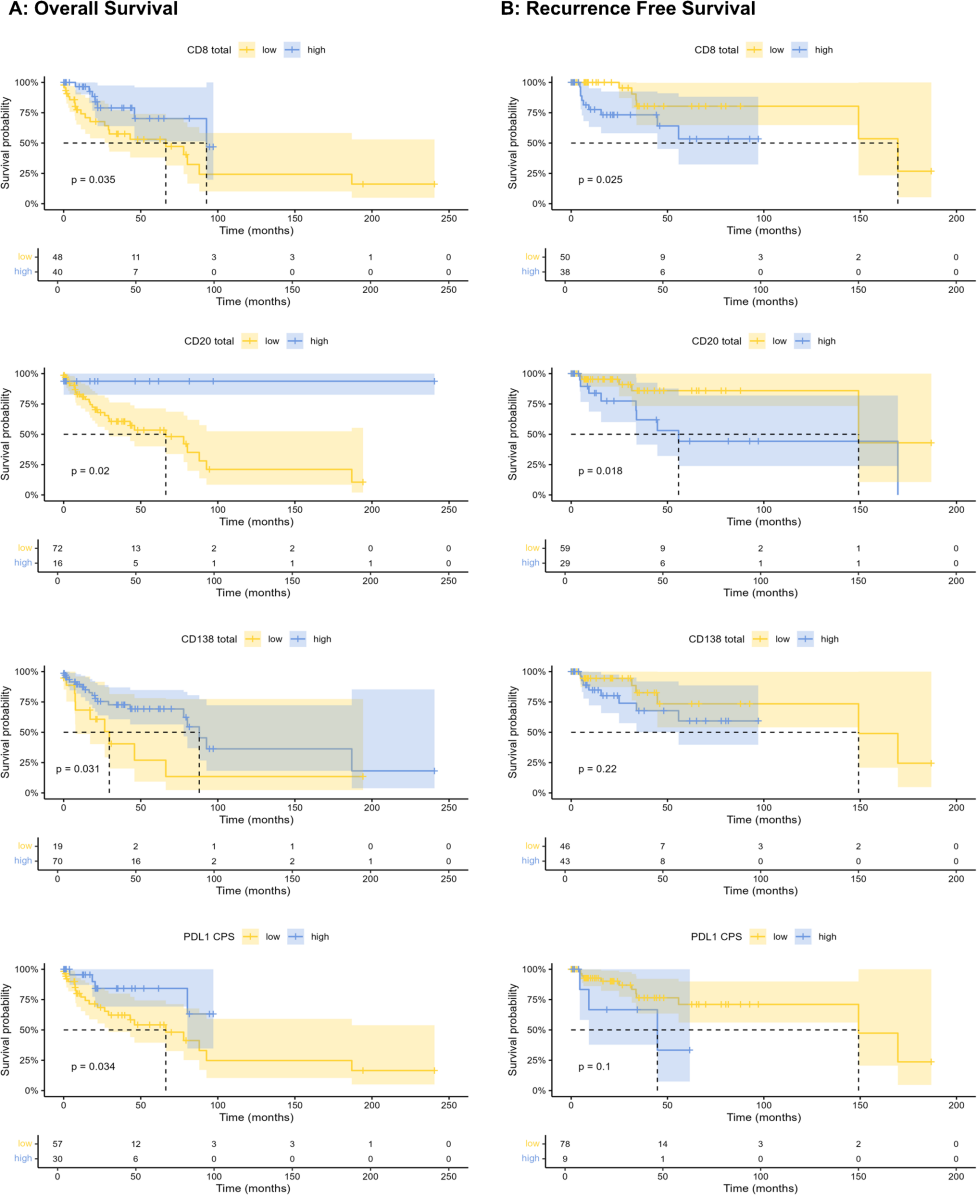
Survival curves of tumor infiltrating immune cells and PD-L1 combined positivity score (CPS) for patients with penile cancer. The densities of CD8 + cytotoxic T cells (CTL), CD20 + B cells, CD138 + plasma cells in the tumor microenvironment, and the PD-L1 CPS were quantified for every patient and dichotomized into low and high levels. The survival curves for **A** overall survival and **B** recurrence-free survival were plotted using the Kaplan–Meier method

The patient cohort was dichotomized into PeCa with low and high immune cell infiltration, or PD-L1 expression (Fig. 1 and Supplemental Table 1). High infiltration of CTL, TIL-B, and plasma cells, as well as a high PD-L1 CPS, was significantly associated with prolonged OS (Fig. 2A). Furthermore, high levels of CTL and TIL-B levels were associated with reduced RFS (Fig. 2B). Plasma cells and PD-L1 expression had no prognostic effect on RFS (Fig. 2B). In the extended univariate survival analysis using the Cox proportional hazard model, high PD-L1 CPS (*p* = 0.04), high total T cell infiltration (*p* = 0.03), high total (*p* = 0.04) or stromal (*p* = 0.02) CTL infiltration, high TIL-B infiltration in any compartment (*p* < 0.05), and high total plasma cell infiltration (*p* = 0.04) were associated with favorable OS (Supplemental Table 1). The combination of chemotherapy and radiation was associated with worse OS (*p* = 0.03). In contrast, a high amount of total (*p* = 0.048) or stromal (*p* = 0.04) T cells, of total (*p* = 0.04) or intratumoral (*p* = 0.03) Th cells, of total (*p* = 0.04) or stromal (*p* = 0.01) CTL, and of total (*p* = 0.03) TIL-B or TIL-B in the tumor parenchyma (*p* = 0.047) were associated with unfavorable RFS (Supplemental Table 2). Again, high infiltration of TIL-B in the tumor parenchyma was associated with favorable DSS (*p* = 0.03), whereas lymph node metastases (*p* ≤ 0.01) and radiation therapy (*p* = 0.01) or combined chemotherapy and radiation therapy (*p* = 0.003) were associated with unfavorable DSS (Supplemental Table 1). Next to the established risk factors tumor grading, stage, and lymph node metastasis, the biomarkers PD-L1 CPS and the T cell subtypes Th cells and CTL as well as TIL-B and plasma cells were included in the multivariate survival analysis when significantly associated with survival in the respective univariate survival analysis. Lymph node metastasis was a significant prognostic factor for adverse OS (*p* = 0.003) and DSS in patients with PeCa (*p* = 0.005) (Table 4). In contrast, a high infiltration of total plasma cells was significantly associated with favorable OS in patients with PeCa (*p* = 0.04) (Table 4). Tumor grading and stage, PD-L1 CPS, Th cells, CTL, TIL-B, and B cells in the tumor parenchyma were not significant prognostic factors in multivariate analysis (Table 4).

**Table 4:** Multivariate survival analysis

	*N*	OS			*N*	RFS			*N*	DSS		
		HR	CI (95%)	*p* value		HR	CI (95%)	*p* value		HR	CI (95%)	*p* value
**Tumor grading**												
G3/G4 vs. G1/G2	18 vs. 58	0.8	0.3–2.2	0.7	17 vs. 58	2.2	0.5–9.9	0.3	18 vs. 61	0.5	0.1–2.4	0.4
**Tumor stage**												
T3 vs. T1/T2	25 vs. 51	0.3	0.1–1.2	0.1	24 vs. 51	0.3	0.1–1.8	0.2	25 vs. 54	0.2	0.03–1.8	0.2
**Lymph node** **metastasis**												
pres. vs. abs	22 vs. 54	5.8	1.8–18.3	**0.003**	22 vs. 53	1	0.2–4.9	0.9	22 vs. 57	7.6	1.8–31.5	**0.005**
**PD-L1 CPS**												
High vs. low	28 vs. 48	0.6	0.2–1.9	0.4	NA	NA	NA	NA	NA	NA	NA	NA
**CD8 total**												
High vs. low	36 vs. 40	0.7	0.2–1.9	0.4	32 vs. 43	2.7	0.6–12.3	0.2	NA	NA	NA	NA
**CD20 total**												
High vs. low	15 vs. 61	0.2	0.02–1.4	0.1	24 vs. 51	1.5	0.3–7.3	0.6	NA	NA	NA	NA
**CD138 total**												
High vs. low	63 vs. 13	0.3	0.1–0.95	**0.04**	NA	NA	NA	NA	NA	NA	NA	NA
**CD20 tumor**												
High vs. low	NA	NA	NA	NA	NA	NA	NA	NA	42 vs. 37	0.4	0.1–1.8	0.2
**CD4 total**												
High vs. low	NA	NA	NA	NA	35 vs. 40	3.9	0.7–21.9	0.1	NA	NA	NA	NA

*OS* overall survival, *RFS* recurrence-free survival, *DSS* disease-specific survival, *HR* hazard’s ratio, *N* number, *CI* confidence interval, *pres*. present, *abs*. absent, *CPS* combined proportion score, *NA* not analyzed

## Discussion

The present study analyzed the prognostic impact of tumor-infiltrating immune cells and PD-L1 expression in patients with PeCa. To this end, tumor-infiltrating immune cells in the tumor parenchyma and associated stroma and PD-L1 status (TPS, CPS, IC) were quantified, and the results were correlated with patient survival and clinicopathological parameters. The main finding of this study was that high densities of plasma cells were associated with favorable OS (70% reduction in mortality risk) for patients with PeCa in the multivariate survival analysis (Table 4). Thereby, in this study, plasma cells were superiorly associated with the outcome compared to established prognostic factors, such as tumor stage or grading. Plasma cells are terminally differentiated B cells and exert their immunological functions mainly through the secretion of antibodies of either the IgM-, IgG-, or the IgA-subtype [13]. After recognizing their tumor-specific antigen and binding to the surface of tumor cells, the antibodies can induce a plethora of different effects, such as antibody-dependent cell-mediated cytotoxicity or phagocytosis, but also complement activation [14]. This plethora of different effects contributes to both anti- and pro-tumor immunity. Plasma cells, when assessed with CD138, are associated with favorable prognosis in colorectal cancer, esophageal cancer, and melanoma, but also with unfavorable prognosis in ovarian cancer, breast cancer, and melanoma [15]. To date, investigations on plasma cells in PeCa, limited to tumors of the usual type only, have not shown any significant prognostic value of this immune cell subtype [16]. In addition to its prognostic value, higher plasma cell levels were observed in the TME of renal cell carcinoma and non-small-cell lung cancer after response to immunotherapy [17]. In bladder cancer, the level of plasma cell infiltration was higher in the group of patients with response to ICI compared to non-responders [18]. This is especially interesting, since the contribution of TIL-B and plasma cells to the response to immunotherapy has recently gained more attention. However, the results on the effectiveness of ICI therapy for patients with PeCa remain inconclusive. Different targeted therapy regimens, including ICI, have been tested for patients with PeCa; however, they were hampered by the rarity of the disease. Three patients with nodal positive PeCa, ineligible for chemotherapy, received therapy with cemiplimab in the first-line setting and showed a partial response (PR) [19]. In a phase 1 study of metastatic genitourinary tumors using the combination of cabozantinib and nivolumab with or without ipilimumab in the second-line setting, three patients with metastatic PeCa were included: one showed PR and two showed stable disease [20]. In a basket trial of HPV-associated carcinomas investigating the effectiveness of a therapeutic HPV vaccine in combination with durvalumab, two patients with PeCa were included, and one of them showed a response to therapy [21]. In a phase 2 trial comparing the combination of radiotherapy and atezolizumab with atezolizumab monotherapy, 32 patients with advanced PeCa were included, and the overall response rate (ORR) was 16.7% [22]. Another study including 92 patients with advanced PeCa and subsequent treatment with ICI reported an ORR of 13% [23]. To date, microsatellite instability, tumor mutational burden [24], and HPV infection [25] are promising candidates as predictive biomarkers for ICI in PeCa to stratify patients with benefit from this treatment. Along with the findings in the present study, it is interesting that tertiary lymphoid structures (TLS), which mainly consist of B cells and mimic the architecture of lymph nodes and other secondary lymphoid organs, are associated with a better response to ICI [26]. TLS provide a niche for immune cell priming and recovery close to the tumor, thereby driving and maintaining effective anti-tumor activity [27]. Taken together, the present study sheds new light on the role of plasma cells in patients with PeCa. These findings should be validated on larger patient cohorts. Further research in the field of immunotherapy should focus on plasma cells, TIL-B, and TLS.

Next to a high infiltration of plasma cells, high densities of CTL and TIL-B were also prognostic for favorable OS and in the case of TIL-B and CTL also for adverse RFS in the univariate analysis (Fig. 2, Supplemental Table 1). However, these associations were not significant in multivariate analysis (Table 4). Therefore, the robustness of these results should be validated in further detail. In our study, the presence of Treg was not prognostic for patient outcomes (Supplemental Table 2). This is in contrast to another study which found a high infiltration of Treg to be an independent prognostic factor for unfavorable disease-free survival [16]. However, differences in the study design might explain the discrepant results, e.g., inclusion of PeCa of the usual type only vs. several PeCa subtypes and analysis of highly inflamed tumor areas only vs. several tumor areas to overcome intratumoral heterogeneity.

For further interpretation of the PD-L1 results, a literature search on PD-L1 analyses on PeCa was undertaken and revealed a heterogeneous landscape of methodologies, used PD-L1 antibodies, definitions of PD-L1 positivity and PD-L1 cutoffs [28–39]. Table 5 provides an overview. The cutoffs used for the dichotomization of the study cohort in PeCa with low and high PD-L1 status and the positivity rate are comparable to the relevant literature (Table 5). In our study, a high PD-L1 CPS was significantly associated with favorable OS in the univariate analysis (Supplemental Table 2), but not in the multivariate (Table 4). So far, the other studies reported either an association with adverse survival or no significant prognostic impact. However, a high number of tumor associated immune cells was associated with prolonged OS and increased PD-L1 CPS [36]. This association leads to the assumption that a high PD-L1 CPS is a surrogate marker for an immune cell-rich TME in patients with PeCa, which is crucial for anti-tumor immunity. In contrast, another study using PD-L1 CPS reported adverse prognostic effects of PD-L1 in patients with PeCa [37]. Taken together, the prognostic and predictive values of PD-L1 in patients with PeCa remain inconclusive which calls for a harmonized consensus of PD-L1 evaluation in PeCa.

**Table 5:** Literature on PD-L1 in penile carcinoma

1st author	Year of publication	Number of patients	Methodology	PD-L1 antibody	PD-L1 analysis	PD-L1 cutoff	PD-L1 positivity rate	Survival	PD-L1 prognostic value
Udager [28]	2016	37	WSI	Merck (clone 5H1)	Membranous staining of tumor cells, presence or absence of PD-L1 staining in TIL	TPS > 5% TIL > 5%	62.20%	CSS	High PD-L1 significantly associated with adverse survival (univariate analysis) multivariate analysis not tested
Deng [29]	2017	116	WSI	Cell Signaling (clone E1L3N)	Membranous staining of tumors cells and TIL	TPS > 5% TIL > 5%	53.4% tumor cells 57% TIL	CSS	High PD-L1 significantly associated with adverse survival (univariate analysis) multivariate analysis ns
Cocks [30]	2017	53	TMA	Cell Signaling	Any membranous positivity of tumor cells or immune cells	TPS > 0% TIL > 0%	40% tumor cells 26% TIL	OS, CSS	ns
Ottenhof [31]	2018	213	WSI	Cell Signaling	Membranous staining of tumor cells and immune cells	TPS > 1%, diffuse vs. marginal staining; TIL > 0%	hrHPV − : 49.4% tumor cells, 72.2% TIL hrHPV + : 32.7% tumor cells, 65.4% TIL	CSS	High PD-L1 (diffuse) significantly associated with adverse survival (multivariate analysis)
Davidson [32]	2018	222	TMA	Abcam (clone 28.8) Spring Biosciences (clone SP142)	Specific membranous and cytoplasmic staining of tumor cells and TIL	TPS and TIL 1 + : < 5%; 2 + : > 5- < 50%; 3 + : > 50%	28.8: 32.1% tumor cells, 64.2% TIL SP142: 7.3% tumor cells, 45% TIL	CSS	High PD-L1 significantly associated with adverse survival (multivariate analysis)
DeBacco [33]	2019	35	WSI	Zeta Corporation (clone ZR3)	Specific membranous and cytoplasmic staining of tumor cells	TPS > 1%	51.40%	OS	ns
Chu [34]	2020	178	WSI	Cell signaling	Membranous staining of tumor cells and immune cells	TPS > 1%, diffuse vs. marginal staining; TIL > 0%	67.4% tumor cells 54.8% TIL	CSS	High PD-L1 (diffuse) significantly associated with adverse survival (univariate analysis) multivariate analysis not tested
Hu [35]	2020	84	WSI	Abcam	H Score	118.79	69%	CSS	High PD-L1 significantly associated with adverse survival (univariate analysis) multivariate analysis ns
Müller [36]	2022	60	WSI	Zeta Corporation	TPS, CPS, IC	0; > 0–10; > 10	TPS 87% IC 83%	OS	ns
Lobo [37]	2023	134	WSI	Cell signaling	CPS	< 1; 1–19; > 20	57%	OS, CSS	High PD-L1 significantly associated with adverse survival (univariate analysis) multivariate analysis not tested
Sangkhamanon [38]	2023	43	WSI	Ventana (clone SP263)	Membranous staining of tumor cells and immune cells	TPS ≥ 25% or TIL > 1% and PD-L1 + ≥ 25% or TIL = 1% and PD-L1 + = 100%	18.60%	OS	ns
Hrudka [39]	2023	165	WSI	Ventana	TPS	< 1%; 1–49%; ≥ 50%	68%	OS, CSS	ns
Present study		93	TMA	Abcam	TPS, CPS, IC	OS: TPS 1%, CPS 3, IC 1% CSS: TPS 0.5%, CPS 6, IC 1% RFS: TPS 2.5%, CPS 15, IC 0.3%	TPS 34.4% CPS 53.3% IC 36.7%	OS, CSS, RFS	High PD-L1 positivity (CPS) significantly associated with better OS (univariate analysis) multivariate ns

*WSI* whole slide image, *TMA* tissue microarray, *TPS* tumor proportion score, *TIL* tumor-infiltrating lymphocytes, *CPS* combined positivity score, *IC* immune cell score, *hrHPV* high-risk human papilloma virus, *CSS* cancer-specific survival, *OS* overall survival, *RFS* remission-free survival, *ns* non-significant

In this study, 25% of the patients were HPV-positive, and HPV infection was not a significant factor for patient outcomes. However, HPV-positive PeCa were associated with a significantly higher infiltration of NK cells and a significantly higher PD-L1 IC score than HPV-negative tumors (Table 3). A higher, yet non-significant, NK cell infiltration in HPV-positive PeCa has been previously demonstrated [40]. The association with the PD-L1 IC score might again be a correlate for more abundant immune cell infiltration in HPV-positive PeCa, as has been shown previously [40]. In general, HPV status can differentiate PeCa into two different pathways of carcinogenesis with different molecular alterations and implications for prognosis and therapy [41]. In the present study, HPV status had no prognostic effect (Supplemental Table 1) or any significant association with TIL-B or CTL (Table 3). Interestingly, pre-existing adiposity was associated with significantly increased TIL-B and Treg levels in the tumor (Table 3). In a murine model, obesity alters metabolism in the tumor microenvironment, thereby promoting tumor growth and impairing CTL responses [42]. Adiposity is also correlated with B cell dysfunction, resulting in enhanced autoimmunity and reduced antibody production [43]. Thus, the increased levels of TIL-B in PeCa of adipose patients (Table 3) are not necessarily a sign of enhanced anti-tumor immunity. The association between TIL-B, obesity, and PeCa and the implications for prognosis and therapy need to be investigated in further detail.

The limitations of this study are its retrospective design, limited sample size, varying duration of FFPE tissue storage, and a bias in the analysis on the prognostic value for DSS through the low number of tumor-specific deaths in this study cohort.

## Conclusion

In summary, in the current study, plasma cells provide superior information on the prognosis of patients with PeCa compared to standard prognostic factors, such as tumor stage or grade. These findings should be validated in further detail using larger patient cohorts. In general, research on the TME of PeCa should focus on plasma cells and TIL-B with respect to response prediction to immunotherapy.

## Supplementary Information

Below is the link to the electronic supplementary material.

Supplementary file1 (DOCX 30 KB)

## Data Availability

All data generated or analyzed during this study are included in this article. Further enquiries can be directed to the corresponding author.
