# Supplementary material for: Tumor-infiltrating plasma cells are a prognostic factor in penile squamous cell carcinoma
Source: Virchows Arch. 2025 Jan 14;487(3):687–99. doi: 10.1007/s00428-024-04013-1 (PMC12488782; doi:10.1007/s00428-024-04013-1)
Supplement: Supplementary file 1 — Supplementary file1 (DOCX 30 KB) [file 428_2024_4013_MOESM1_ESM.docx]

| **Supplemental Table 1: Cutoff values for dichotomization of** | | | | |
| --- | --- | --- | --- | --- |
| **the patient cohort.** | |  |  |  |
|  |  |  |  |  |
| **Biomarker** | **Compartment** | **Cutoff OS** | **Cutoff RFS** | **Cutoff DSS** |
|  | TPS | 1.0 | 2.5 | 0.5 |
| PD-L1 | CPS | 3.0 | 15.0 | 6.0 |
|  | IC | 1.0 | 0.3 | 1.0 |
|  | tumor | 687.3 | 733.4 | 89.6 |
| CD3 | stroma | 3127.2 | 1909.1 | 3090.3 |
|  | total | 1263.4 | 541.4 | 1144.1 |
|  | tumor | 133.4 | 50.2 | 50.2 |
| CD4 | stroma | 32.3 | 5.0 | 337.3 |
|  | total | 321.5 | 110.0 | 236.5 |
|  | tumor | 225.9 | 273.8 | 159.2 |
| CD8 | stroma | 838.4 | 778.7 | 84.0 |
|  | total | 303.4 | 310.1 | 1230.9 |
|  | tumor | 3.9 | 0.0 | 1.8 |
| CD20 | stroma | 341.8 | 147.7 | 341.8 |
|  | total | 159.4 | 66.6 | 159.4 |
|  | tumor | 11.4 | 6.3 | 5.5 |
| CD56 | stroma | 29.3 | 12.1 | 29.3 |
|  | total | 16.8 | 6.3 | 9.7 |
|  | tumor | 0.7 | 5.7 | 4.3 |
| CD138 | stroma | 3.9 | 1846.8 | 1643.9 |
|  | total | 2.2 | 26.0 | 1.5 |
|  | tumor | 10.9 | 0.5 | 3.7 |
| FoxP3 | stroma | 51.5 | 1.6 | 91.5 |
|  | total | 19.8 | 2.4 | 19.8 |
| OS: overall survival; RFS: recurrence free survival; DSS: disease specific survival; TPS: tumor proportion score; CPS: combined positivity score; IC: immune cell score | | | | |

| **Supplemental Table 2: Univariate Survival Analysis.** | | | | | |  |  |  |  |  |  |  |  |
| --- | --- | --- | --- | --- | --- | --- | --- | --- | --- | --- | --- | --- | --- |
|  |  |  |  |  |  |  |  |  |  |  |  |  |  |
|  |  |  | **OS** | | |  | **RFS** | | |  | **DSS** | | |
| **Biomarker** | **Group** | **N** | **HR** | **CI (95%)** | **p value** | **N** | **HR** | **CI (95%)** | **p value** | **N** | **HR** | **CI (95%)** | **p value** |
| **PD-L1 TPS** | low | 61 | ref |  |  | 64 | ref |  |  | 60 | ref |  |  |
|  | high | 26 | 0.4 | 0.1-1.2 | 0.09 | 23 | 1.8 | 0.6-5.5 | 0.3 | 27 | 0.7 | 0.2-2.6 | 0.6 |
| **PD-L1 CPS** | low | 57 | ref |  |  | 78 | ref |  |  | 63 | ref |  |  |
|  | high | 30 | 0.3 | 0.1-0.97 | **0.04** | 9 | 2.8 | 0.8-10.2 | 0.1 | 24 | 0.5 | 0.1-2.5 | 0.4 |
| **PD-L1 IC** | low | 72 | ref |  |  | 61 | ref |  |  | 72 | ref |  |  |
|  | high | 15 | 0.0 | 0-inf | 1.0 | 26 | 0.4 | 0.1-1.7 | 0.2 | 15 | <0.001 | 0-inf | 1.0 |
| **CD3 Total** | low | 58 | ref |  |  | 27 | ref |  |  | 53 | ref |  |  |
|  | high | 25 | 0.3 | 0.1-0.9 | **0.03** | 56 | 8.0 | 1.0-62.3 | **0.048** | 30 | 0.3 | 0.1-1.4 | 0.1 |
| **CD3 Tumor** | low | 55 | ref |  |  | 57 | ref |  |  | 8 | ref |  |  |
|  | high | 28 | 0.4 | 0.2-1.2 | 0.1 | 26 | 2.5 | 0.8-7.6 | 0.10 | 75 | 0.3 | 0.1-1.6 | 0.2 |
| **CD3 Stroma** | low | 68 | ref |  |  | 49 | ref |  |  | 65 | ref |  |  |
|  | high | 15 | 0.3 | 0.1-1.2 | 0.09 | 34 | 3.5 | 1.1-11.0 | **0.04** | 18 | 0.3 | 0.1-2.2 | 0.2 |
| **CD4 Total** | low | 67 | ref |  |  | 47 | ref |  |  | 62 | ref |  |  |
|  | high | 17 | 0.4 | 0.1-1.4 | 0.2 | 37 | 3.5 | 1.1-11.2 | **0.04** | 22 | 3.2 | 1.0-10.4 | 0.1 |
| **CD4 Tumor** | low | 62 | ref |  |  | 49 | ref |  |  | 49 | ref |  |  |
|  | high | 22 | 0.5 | 0.2-1.5 | 0.2 | 35 | 3.7 | 1.2-11.9 | **0.03** | 35 | 2.2 | 0.6-7.4 | 0.2 |
| **CD4 Stroma** | low | 19 | ref |  |  | 8 | ref |  |  | 48 | ref |  |  |
|  | high | 64 | 0.6 | 0.3-1.3 | 0.2 | 75 | >10 | 0-inf | 1.0 | 35 | 3.0 | 0.8-11.3 | 0.1 |
| **CD8 Total** | low | 48 | ref |  |  | 50 | ref |  |  | 78 | ref |  |  |
|  | high | 40 | 0.4 | 0.2-0.96 | **0.04** | 38 | 3.6 | 1.1-11.6 | **0.04** | 10 | <0.001 | 0-inf | 1.0 |
| **CD8 Tumor** | low | 50 | ref |  |  | 56 | ref |  |  | 47 | ref |  |  |
|  | high | 37 | 0.5 | 0.2-1.2 | 0.1 | 31 | 3.1 | 0.9-9.6 | 0.1 | 40 | 2.3 | 0.7-7.9 | 0.2 |
| **CD8 Stroma** | low | 59 | ref |  |  | 55 | ref |  |  | 10 | ref |  |  |
|  | high | 26 | 0.2 | 0.04-0.7 | **0.02** | 30 | 4.5 | 1.3-15.0 | **0.01** | 75 | 0.3 | 0.1-1.2 | 0.1 |
| **CD20 Total** | low | 72 | ref |  |  | 59 | ref |  |  | 72 | ref |  |  |
|  | high | 16 | 0.1 | 0.02-0.98 | **0.048** | 29 | 3.5 | 1.2-10.5 | **0.03** | 16 | <0.001 | 0-inf | 1.0 |
| **CD20 Tumor** | low | 55 | ref |  |  | 25 | ref |  |  | 41 | ref |  |  |
|  | high | 33 | 0.3 | 0.1-0.9 | **0.02** | 63 | 8.0 | 1.0-62.6 | **0.047** | 47 | 0.2 | 0.1-0.8 | **0.03** |
| **CD20 Stroma** | low | 69 | ref |  |  | 59 | ref |  |  | 69 | ref |  |  |
|  | high | 17 | 0.1 | 0.02-0.9 | **0.04** | 27 | 2.3 | 0.8-7.0 | 0.1 | 17 | <0.001 | 0-inf | 1.0 |
| **CD56 Total** | low | 80 | ref |  |  | 75 | ref |  |  | 78 | ref |  |  |
|  | high | 9 | 0.3 | 0.05-2.5 | 0.3 | 14 | 2.1 | 0.6-7.7 | 0.3 | 11 | 0.6 | 0.1-4.9 | 0.7 |
| **CD56 Tumor** | low | 80 | ref |  |  | 78 | ref |  |  | 77 | ref |  |  |
|  | high | 10 | 0.4 | 0.1-2.8 | 0.4 | 12 | 1.3 | 0.3-5.9 | 0.7 | 13 | 0.5 | 0.1-3.7 | 0.5 |
| **CD56 Stroma** | low | 79 | ref |  |  | 75 | ref |  |  | 79 | ref |  |  |
|  | high | 9 | 1.4 | 0.4-4.8 | 0.6 | 14 | 1.4 | 0.3-6.6 | 0.6 | 9 | 1.8 | 0.4-8.3 | 0.5 |
| **CD138 Total** | low | 70 | ref |  |  | 46 | ref |  |  | 17 | ref |  |  |
|  | high | 19 | 0.4 | 0.2-0.9 | **0.04** | 43 | 2.0 | 0.6-6.1 | 0.2 | 72 | 0.4 | 0.1-1.4 | 0.2 |
| **CD138 Tumor** | low | 65 | ref |  |  | 79 | ref |  |  | 76 | ref |  |  |
|  | high | 23 | 1.4 | 0.7-3.1 | 0.4 | 9 | 0.0 | 0-inf | 1.0 | 12 | 0.5 | 0.1-4.2 | 0.6 |
| **CD138 Stroma** | low | 15 | ref |  |  | 74 | ref |  |  | 71 | ref |  |  |
|  | high | 69 | 0.5 | 0.2-1.1 | 0.1 | 10 | 3.2 | 0.9-11.8 | 0.1 | 13 | <0.001 | 0-inf | 1.0 |
| **FoxP3 Total** | low | 41 | ref |  |  | 8 | ref |  |  | 41 | ref |  |  |
|  | high | 48 | 0.5 | 0.3-1.2 | 0.1 | 81 | 4.2 | 0.5-35.8 | 0.2 | 48 | 0.4 | 0.1-1.2 | 0.1 |
| **FoxP3 Tumor** | low | 49 | ref |  |  | 11 | ref |  |  | 27 | ref |  |  |
|  | high | 40 | 0.6 | 0.3-1.3 | 0.2 | 78 | 4.5 | 0.5-37.6 | 0.2 | 62 | 5.3 | 0.7-41.2 | 0.1 |
| **FoxP3 Stroma** | low | 34 | ref |  |  | 8 | ref |  |  | 50 | ref |  |  |
|  | high | 55 | 0.7 | 0.3-1.4 | 0.3 | 81 | 4.1 | 0.5-35.1 | 0.2 | 39 | 0.4 | 0.1-1.6 | 0.2 |
| **Histologic Subtype** | basaloid | 15 | ref |  |  | 15 | ref |  |  | 15 | ref |  |  |
|  | usual type | 74 | 0.6 | 0.3-1.6 | 0.3 | 74 | 0.9 | 0.2-3.2 | 0.8 | 74 | 0.8 | 0.2-3.8 | 0.8 |
| **Tumor Grading** | G1 | 17 | ref |  |  | 17 | ref |  |  | 17 | ref |  |  |
|  | G2 | 53 | 2.0 | 0.6-6.8 | 0.3 | 53 | 0.6 | 0.2-2.2 | 0.5 | 53 | 3.1 | 0.4-24.7 | 0.3 |
|  | G3 | 19 | 3.7 | 1.0-13.9 | 0.1 | 19 | 0.9 | 0.2-3.9 | 0.9 | 19 | 3.5 | 0.4-33.5 | 0.3 |
|  | G4 | 2 | 1.3 | 0.1-12.5 | 0.8 | 2 | 0.0 | 0-inf | 1.0 | 2 | <0.001 | 0-inf | 1.0 |
| **Tumor Stage** | pT1 | 35 | ref |  |  | 35 | ref |  |  | 35 | ref |  |  |
|  | pT2 | 27 | 1.2 | 0.5-2.6 | 0.7 | 27 | 1.4 | 0.4-4.6 | 0.6 | 27 | 0.8 | 0.2-3.0 | 0.8 |
|  | pT3 | 27 | 0.4 | 0.1-1.40 | 0.2 | 27 | 0.7 | 0.2-3.1 | 0.7 | 27 | 0.2 | 0.03-2.0 | 0.2 |
| **Lymph Node Metastasis** | pN0/N0/Nx | 60 | ref |  |  | 60 | ref |  |  | 60 | ref |  |  |
|  | pN1/N1 | 7 | 3.1 | 0.8-11.5 | 0.1 | 7 | 3.4 | 0.7-16.6 | 0.1 | 7 | 6.2 | 1.03-37.6 | **0.046** |
|  | pN2/N2 | 6 | 6.4 | 1.9-22.1 | **0.003** | 6 | 2.5 | 0.3-22.9 | 0.4 | 6 | 19.2 | 4.0-93.3 | **<0.001** |
|  | pN3/N3 | 9 | 1.6 | 0.4-7.5 | 0.5 | 9 | 0.0 | 0-inf | 1.0 | 9 | 5.2 | 0.9-31.4 | 0.07 |
| **Distant Metastasis** | M0 | 24 | ref |  |  | 24 | ref |  |  | 24 | ref |  |  |
|  | M1 | 5 | 2.3 | 0.4-11.9 | 0.3 | 5 | 1.5 | 0.2-13.7 | 0.7 | 5 | 3.7 | 0.6-22.4 | 0.2 |
| **Resection Status** | R0 | 65 | ref |  |  | 65 | ref |  |  | 65 | ref |  |  |
|  | R1 | 6 | 0.0 | 0-inf | 1.0 | 6 | 0.0 | 0-inf | 1.0 | 6 | <0.001 | 0-inf | 1.0 |
| **Primary Tumor Surgery** | Partial Penectomy | 55 | ref |  |  | 55 | ref |  |  | 55 | ref |  |  |
|  | Total Penectomy | 25 | 1.1 | 0.5-2.4 | 0.9 | 25 | 1.0 | 0.3-3.4 | 1.0 | 25 | 2 | 0.6-6.4 | 0.3 |
|  | Tumor Excision | 4 | 0.0 | 0-inf | 1.0 | 4 | 2.2 | 0.4-11.1 | 0.4 | 4 | <0.001 | 0-inf | 1.0 |
|  | Circumcision | 8 | 0.4 | 0.1-3.2 | 0.4 | 8 | 1.1 | 0.1-8.7 | 1.0 | 8 | <0.001 | 0-inf | 1.0 |
| **Subsequent Therapy** | None | 67 | ref |  |  | 67 | ref |  |  | 67 | ref |  |  |
|  | CTX | 16 | 1.1 | 0.4-2.8 | 0.8 | 16 | 1.3 | 0.4-4.1 | 0.7 | 16 | 2.9 | 0.8-10.7 | 0.1 |
|  | Radiatio | 1 | 7.9 | 1.0-63.2 | 0.05 | 1 | 0.0 | 0-inf | 1.0 | 1 | 18.9 | 2.0-178.6 | **0.01** |
|  | Unknown | 6 | 0.8 | 0.2-3.4 | 0.8 | 6 | 0.8 | 0.1-6.1 | 0.8 | 6 | <0.001 | 0-inf | 1.0 |
|  | CTX and Radiatio | 2 | 5.3 | 1.2-24.0 | **0.03** | 2 | 0.0 | 0-inf | 1.0 | 2 | 14.1 | 2.5-78.6 | **0.003** |
| **HPV infection** | absent | 68 | ref |  |  | 68 | ref |  |  | 68 | ref |  |  |
|  | present | 24 | 0.8 | 0.3-2.0 | 0.7 | 24 | 1.4 | 0.5-4.2 | 0.5 | 24 | 0.7 | 0.1-3.2 | 0.6 |
| **Diabetes mellitus** | absent | 74 | ref |  |  | 74 | ref |  |  | 74 | ref |  |  |
|  | present | 19 | 1.9 | 0.8-4.5 | 0.1 | 19 | 0.9 | 0.2-4.1 | 0.9 | 19 | 0.8 | 0.2-3.5 | 0.7 |
| **Adipositas** | absent | 79 | ref |  |  | 79 | ref |  |  | 79 | ref |  |  |
|  | present | 14 | 1.1 | 0.4-2.8 | 0.8 | 14 | 0.8 | 0.2-3.8 | 0.8 | 14 | 1.2 | 0.3-5.6 | 0.8 |
| **Smoking** | absent | 70 | ref |  |  | 70 | ref |  |  | 70 | ref |  |  |
|  | present | 23 | 1.8 | 0.8-3.7 | 0.1 | 23 | 1.1 | 0.4-3.2 | 0.9 | 23 | 2.9 | 1.0-9.0 | 0.1 |
| **Phimosis** | absent | 68 | ref |  |  | 68 | ref |  |  | 68 | ref |  |  |
|  | present | 25 | 0.9 | 0.4-2.0 | 0.7 | 25 | 0.5 | 0.1-1.7 | 0.3 | 25 | 1.5 | 0.4-4.9 | 0.5 |
| **Balanoposthitis** | absent | 83 | ref |  |  | 83 | ref |  |  | 83 | ref |  |  |
|  | present | 10 | 0.5 | 0.1-2.3 | 0.4 | 10 | 1.6 | 0.4-6.1 | 0.5 | 10 | 1 | 0.1-7.7 | 1.0 |
| **Condyloma accuminata** | absent | 81 | ref |  |  | 81 | ref |  |  | 81 | ref |  |  |
|  | present | 3 | 1.2 | 0.2-9.3 | 0.8 | 3 | 0.0 | 0-inf | 1.0 | 3 | <0.001 | 0-inf | 1.0 |
| **Lichen sclerosus** | absent | 88 | ref |  |  | 88 | ref |  |  | 88 | ref |  |  |
|  | present | 5 | 1.1 | 0.1-8.0 | 1.0 | 5 | 2.2 | 0.3-16.7 | 0.5 | 5 | <0.001 | 0-inf | 1.0 |
| OS: overall survival; RFS: recurrence free survival; DSS: disease specific survival; HR: hazard's ratio; N: number | | | | | | | | | | | | | |
| CI: confidence intervall; TPS: tumor proportion score; CPS: combined proportion score; IC: immune cell score | | | | | | | | | | | | |  |
